# Curcumin and tetrahydrocurcumin both prevent osteoarthritis symptoms and decrease the expressions of pro-inflammatory cytokines in estrogen-deficient rats

**DOI:** 10.1186/s12263-016-0520-4

**Published:** 2016-03-17

**Authors:** Sunmin Park, La Ra Lee, Ji Hyun Seo, Suna Kang

**Affiliations:** Department of Food and Nutrition, College of Natural Sciences, Obesity/Diabetes Center, Hoseo University, 165 Sechul-Ri, BaeBang-Yup, Asan-Si, ChungNam-Do 336-795 South Korea

**Keywords:** Curcumin, Tetrahydrocurcumin, Bone mineral density, Lean body mass, Estrogen deficiency, Monoiodoacetate

## Abstract

**Background:**

Menopausal symptoms are associated with inflammation. Curcumin is a well-known anti-inflammatory bioactive compound from turmeric whereas tetrahydrocurcumin (THC) is a major metabolite of curcumin that may have different efficacies. However, they have not been studied for anti-menopausal symptoms and anti-osteoarthritis effects. We compared the efficacies of curcumin and THC for preventing postmenopausal and osteoarthritis symptoms in ovariectomized (OVX) obese rats with monoiodoacetate (MIA) injections into the right knee to generate a similar pathology as osteoarthritis.

**Methods:**

OVX rats were provided a 45 % fat diet containing either (1) 0.4 % curcumin (curcumin), (2) 0.4 % THC, (3) 30 μg/kg body weight 17β-estradiol + 0.4 % dextrin (positive control), (4) 0.4 % dextrin (placebo; control), or (5) 0.4 % dextrin with no MIA injection (normal control) for 4 weeks. At the beginning of the fifth week, OVX rats were given articular injections of MIA or normal-control saline into the right knee and the assigned diets were provided for an additional 3 weeks.

**Results:**

Curcumin and THC had similar efficacies for skin tail temperature in OVX rats whereas THC, but not curcumin, prevented glucose intolerance, which might be involved in exacerbating osteoarthritis. Both protected against osteoarthritis symptoms and pain-related behaviors better than 17β-estradiol treatment in estrogen-deficient rats. Curcumin and THC prevented the deterioration of articular cartilage compared to control. They also maintained lean body mass and lowered fat mass as much as 17β-estradiol treatment. The improvement in osteoarthritis symptoms was associated with decreased gene expressions of matrix metalloproteinase (*MMP*)*3* and *MMP13* and tumor necrosis factor-α, interleukin (*IL*)*1β*, and *IL6* in the articular cartilage.

**Conclusions:**

THC and curcumin are effective for treating postmenopausal and osteoarthritis symptoms in OVX rats with MIA-induced osteoarthritis-like symptoms and may have potential as interventions for menopausal and osteoarthritic symptoms in humans.

## Background

Osteoarthritis (OA) is a degenerative joint disease that is characterized by a slow deterioration of the joints. OA is a leading cause of physical disability, and its incidence is rising due to increasing obesity and an aging population (Johnson and Hunter 2014). Both are associated with systemic inflammation (Greene and Loeser 2015). The prevalence of knee OA increased 26.6 % from 1990 to 2010 in 21 epidemiological regions worldwide (Vos et al. 2012). The occurrence of OA increases with age due to the decreased capacity to suppress inflammation, age-related sarcopenia, and increased bone turnover (Johnson and Hunter 2014). Women experience a higher prevalence and severity of OA in the hands, feet, and knees than men and are more often affected with OA than men (Srikanth et al. 2005). In women, the risk of OA especially increases at menopause and estrogen protects against OA (Hanna et al. 2004). The gender disparities in OA may be related to multiple factors including the following: pain sensitivity, lower volume of knee cartilage, bone strength, alignment, ligament laxity, pregnancy, and neuromuscular strength (Johnson and Hunter 2014). In addition, the dysregulation of glucose and energy metabolism also exacerbates OA symptoms in postmenopausal women (King and Rosenthal 2015; Thijssen et al. 2015). Therefore, postmenopausal women are clearly more susceptible to OA.

There is no effective treatment for OA. Since the exact causes of the disease remain unknown, fundamental drugs have not been developed. OA is induced by the inflammation and catabolism of the joints, which causes severe pain (Houard et al. 2013; Wu et al. 2012). The current OA treatments mostly address joint pain. The existing pharmaceuticals for OA such as analgesics, steroids, and non-steroidal anti-inflammatory drugs reduce the symptoms of OA by reducing pain and inflammation (Suokas et al. 2014). However, their long-term use cannot be sustained due to inadequate pain relief or serious gastrointestinal and cardiovascular adverse events (Schnitzer 2006). Therefore, new treatments for OA with better safety and efficacy are needed.

Curcumin is an active component of turmeric (*Curcuma longa*), which has a long history of safe use in foods. It has long been used as an anti-inflammatory treatment in traditional Chinese and Ayurvedic medicine (Goel et al. 2008). The yellow-pigmented fraction of turmeric contains curcuminoids, which are chemically related to its principal ingredient, curcumin. Curcumin is reported to have beneficial effects on OA, type 2 diabetes, and dyslipidemia. Turmeric extracts change colors according to acidic and alkaline pH, and tetrahydrocurcumin (THC), a major metabolite of curcumin, is whitish colored. THC has also been reported to have anti-oxidant, anti-inflammatory, chemopreventive, anti-bacterial, anti-dyslipidemic, and anti-aging activities (Muthumani and Miltonprabu 2015; Sangartit et al. 2014; Wu et al. 2014; Xiang et al. 2011). Although THC has similar effects, recent studies have shown that THC has superior anti-oxidant properties with increased GSH peroxidase, glutathione-S-transferase, NADPH: quinine reductase, and free radical quenching activities (Aggarwal et al. 2015). In addition, curcumin is stable at pH 1–6 (Wang et al. 1997) but not at neutral pH (about pH 7–8) and loses anti-oxidant capacity. Thus, curcumin may be degraded in the intestines and may not work as an anti-oxidant in vivo. Curcumin which is not degraded in the intestines is metabolized into THC that is quite stable and still possesses anti-oxidant activities at neutral or basic pH (Murugan and Pari 2006) (Somparn et al. 2007). Thus, THC may be more potent in the intestines than curcumin in animals and humans.

The hypothesis of the present study was that the long-term consumption of tetrahydrocurcumin and curcumin would prevent and delay the symptoms of OA in obese animals with estrogen deficiency. The present study tested the hypothesis and explored the nature of their protection against OA in ovariectomized (OVX) rats with OA induced by intra-articular injection of monoiodoacetate (MIA). The MIA injection is known to produce a similar pathology as OA by disrupting glycolysis at the site of the injection resulting in the eventual death of chondrocytes and by increased inflammation (Guzman et al. 2003).

## Methods

### The major components

**Fig. 1:**
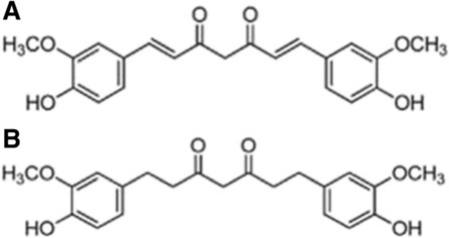
Chemical structure of curcumin and tetrahydrocurcumin. **a** Curcumin, C_21_H_20_O_6_. **b** Tetrahydrocurcumin, C_21_H_24_O_6_

The curcumin extract contained curcumin, demethoxycurcumin, and bismethoxycurcumin at 79.9, 17.0, and 3.1 %, respectively, and the purity of THC was 95.7 %. As determined by HPLC (Sami Labs Limited, Bangalore, India). THC from *C. longa* were generously provided by Sabinsa Corp. (NJ, USA). Their chemical structures are given in Fig. 1.

### Diet preparation

The dosages of curcumin and tetrahydrocurcumin were determined by our preliminary study: each test diet contained 0.4 % curcumin or tetrahydrocurcumin in a high-fat diet. The dosage in the diet was based on our preliminary cell culture study: low dosage (0.5–1.5 μg/mL) treatment of curcumin and tetrahydrocurcumin lowered tumor necrosis factor-α (*TNF-α*) expression in lipopolysaccharide (LPS) activated RAW 264.7 cells (mouse leukaemic monocyte/macrophage cell line).

All groups had high-fat diets that were intended to exacerbate the progression of OA and menopausal symptoms, possibly due to obesity, as compared to a low-fat diet (Lizcano and Guzman 2014; Thijssen et al. 2015). The semi-purified modified AIN-93 diet (Reeves 1997) consisted of 35 % energy (En%) from carbohydrates, 20 En% from protein, and 45 En% from fats. The major carbohydrate, protein, and fat sources were starch plus sugar, casein (milk protein), and lard (CJ Co., Seoul, Korea), respectively. High-fat diets were supplemented with 0.4 % curcumin, tetrahydrocurcumin, or dextrin (control) or 30 μg/kg body weight 17β-estradiol + 0.4 % dextrin (positive control) to make the nutrient composition of the diets the same. Each powder of curcumin and tetrahydrocurcumin was homogeneously mixed with the vitamin and mineral mixture and sugar, and then, the mixture was sifted to break apart lumps. Each mixture was blended with the appropriate amounts of starch, casein, and lard; sifted again; and stored at 4 °C. Every other day, the remaining feed was removed and weighed to measure consumption, and fresh weighed feed was provided. The amount of each supplement consumed (dosage) was calculated from the feed intake.

### Estrogen-deficient and MIA-induced OA animal model

Female Sprague Dawley rats (weighing 231 ± 20 g) were housed individually in stainless steel cages in a controlled environment (23 °C and with a 12-h light/dark cycle). This research followed the guidelines of the NIH Guide for the Care and Use of Laboratory Animals and the International Association for the Study of Pain and was approved by the Animal Care and Use Committee of Hoseo University, Korea (2013-04). After purchasing Sprague Dawley rats from DBL (Yeumsung-Kun, Korea), they were acclimated for 1 week in our animal facility. Rats underwent ovariectomy (OVX) under anesthesia induced by subcutaneous injection of a mixture of ketamine and xylazine (100 and 10 mg/kg body weight, respectively, used for all anesthesia) (Ko et al. 2014). A mid-ventral incision was made, and each ovary was isolated by ligation of the most proximal portion of the oviduct, and then, ovaries were removed with scissors.

After a 4-week treatment with the assigned diets, each OVX rat was then subjected to a single intra-articular injection of MIA (4 mg/50 μL saline; Sigma Co.) through the patellar ligament of the right knee, using a 26-gauge needle after anesthetization (Park et al. 2014). The normal-control group had a single intra-articular injection of saline into the right knee of the OVX rats fed a high-fat diet. On the day following MIA injections, the rats were carefully observed for their posture, behaviors, and development of swelling in the right knee by a trained technician who evaluated all rats.

### Experimental design

The OVX rats were randomly assigned to the following five groups (*n* = 10 each): (1) MIA injection into the knee and 0.4 % curcumin (curcumin), (2) MIA injection into the knee and 0.4 % tetrahydrocurcumin (THC), (3) MINA injection into the knee and 30 μg/kg body weight 17β-estradiol + 0.4 % dextrin (positive control), (4) MIA injection into the knee and 0.4 % dextrin (placebo; control), and (5) saline injection into the knee and 0.4 % dextrin (normal control). After OVX operations, the rats were given free access to water and their respective diets.

After 4 weeks of providing the assigned diets, MIA or saline was injected into the right knee of the OVX rats of each group as described below and the assigned diets were provided for an additional 3 weeks. At the seventh week of the experimental period, an oral glucose tolerance test (OGTT) was performed in overnight feed-deprived rats by orally administering 2 g/kg body weight of glucose every 10 min for 90 and 120 min and serum insulin levels were measured at 0, 20, 40, 60, 90, and 120 min (Ko et al. 2014). Serum glucose and insulin levels were measured using a glucometer (Accuchek, Roche Diagnostics, Indianapolis, IN) and RIA kit (Linco Research, Billerica, MA), respectively. Insulin resistance was determined using the homeostasis model assessment estimate of insulin resistance (HOMA-IR) calculated using the following equation: HOMA-IR = fasting insulin (μIU/ml) × fasting glucose (mM)/22.5. At the end of the study, the rats were anesthetized and peri-uterine and retroperitoneal fat pads and uteri were removed and weighed. Blood samples for serum isolation were collected by cardiac puncture, allowed to coagulate, and serum prepared by centrifugation and then stored at −70 °C for biochemical analysis.

### Tail skin temperature measurement

Tail skin temperature was monitored on the fourth and eighth weeks of the experimental periods during the sleep cycle using an infrared thermometer (BIO-152-IRB, Bioseb, Chaville, France) designed for small rodents. Three measurements were made 10 min apart, and the average value for the animal was used as a single data point.

### BMD measurement

Prior to use, a dual-energy X-ray absorptiometer (DEXA; Norland pDEXA Sabre; Norland Medical Systems Inc., Fort Atkinson, WI, USA) was calibrated with a phantom supplied by the manufacturer. After anesthetization, the animals were laid in a prone position, with the posterior legs maintained in external rotation with tape. The hip, knee, and ankle articulations were in 90° flexion. Upon the completion of scanning, bone mineral density (BMD) was determined in the right femur and knee using the DEXA instrument equipped with the appropriate software for assessment of bone density in small animals (Ko et al. 2014). Similarly, abdominal fat mass and lean mass were measured by DEXA.

### Progression of OA and pain-related behavior tests

At 3, 7, 14, and 21 days after MIA injection, the diameters of the knees were measured every week using digital calipers (Mitotoyo, Japan). For gross observation, all experimental rats were weighed and carefully inspected every week to assess knee joint swelling and gait disturbances under natural conditions in the cages, where they were allowed to move freely. Swelling and limping were classified as no change (0), mild (1), moderate (2), and severe (3) on the basis of severity (Kobayashi et al. 2003). All assessments were conducted by the same trained inspector who was blinded to treatment details throughout the study period.

At 7, 14, and 21 days after MIA injection, pain-related behaviors were assessed by an incapacitance test using a hind paw limb weight-bearing apparatus (Linton Incapacitance Tester, UK) and the maximum running speed on a treadmill. These tests have been utilized as indices of joint discomfort and may be useful for the discovery of novel pharmacologic agents in human OA (Pomonis et al. 2005). The incapacitance tester is a device that compares differences in hind paw weight distribution between the right (osteoarthritic) and left (control) limbs. Animals were acclimated for 30 min before the test. Measurements were performed five times in each rat, and the average of the middle three values was calculated. Percent weight distribution of the right hind paw was calculated using the following formula: % weight distribution of right hind paw = right weight/(left weight + right weight) × 100 (Bar-Yehuda et al. 2009). Since animals with OA cannot walk or run as fast as normal animals, the maximum running speed can be a diagnostic criterion for OA. Rats were acclimated to the treadmill at 40 cm/s for 1 min and then increased to 50 cm/s for 1 min. Subsequently, the speed of the treadmill was increased by 5 cm/s at 1 min intervals until the rats could not continue running and slid into the back of the treadmill. The maximum speed for running by rats was defined as the fastest speed the rats could maintain for 20 s. All experimental rats were subjected to the treadmill test for less than 5 min in each experiment.

### Isolation of total RNA from articular cartilage and real-time PCR

Articular cartilage samples from five rats of each group were collected 28 days after MIA injection, and each cartilage was individually powdered with a cold steel mortar and pestle and then mixed with a monophasic solution of phenol and guanidine isothiocyanate (TRIzol reagent, Life Technologies, Rockville, MD, USA) for total RNA extraction, following the manufacturer’s instructions. RNA concentrations were determined using a Lamda 850 spectrophotometer (Perkin Elmer, Waltham, MA, USA). The cDNA was synthesized from 1 μg total RNA extracted from each rat using a superscript III reverse transcriptase kit (Life Science Technology). Five different cDNA transcripts were made from five rats from each group and were used for real-time PCR. The cDNA from each rat and primers for specific genes were mixed with SYBR Green mix (Bio-Rad, Richmond, CA) in duplicate and amplified using a real-time PCR instrument (Bio-Rad). Thermal cycling conditions were 55 °C for 2 min, 95 °C for 10 min followed by 40 cycles of 94 °C for 20 s, 65 °C for 30 s, and 72 °C for 20 s. To assess genes associated with the inflammation and degradation of articular cartilage, primers of *TNF-α*, interleukin (*IL*)*1β*, *IL6*, matrix metalloproteinase (*MMP*)*3*, and *MMP13* genes were used as described previously (Ko et al. 2014; Park et al. 2014). The gene expression levels in unknown samples were quantitated using the comparative cycle of threshold (CT) method (ΔΔCT method) (Livak and Schmittgen 2001). ΔCT was calculated using the formula: ΔCT = CT (target gene) − CT (endogenous reference gene, β-actin). The relative fold-change in expression was calculated by the equation of ΔΔCt = ΔCt_treatment_ − ΔCt_control_. Results were presented as 2^−ΔΔCT^.

### Histopathological analysis of knee

After 21 days following MIA injection, rats were sacrificed and histologically examined to assess chronic morphological changes in the knee articular bones for narrowing, loss of joint region, cartilage erosion, and osteophyte formation (Bar-Yehuda et al. 2009; Guzman et al. 2003; Park et al. 2014). For histological analysis, knee joints were removed, fixed in phosphate-buffered formalin, decalcified in 10 % nitric acid for 72 h, and embedded in paraffin. Five-micrometer sections were stained with hematoxylin and eosin (H-E) and safranin-O fast green, and morphological changes were observed. Histopathological changes in each animal were quantitatively expressed by the following scoring system (Guzman et al. 2003). Cartilage damage was evaluated according to the depth and extent of the damage on a scale of 0–5 where 0 was normal; 1, minimal, affecting the superficial zone only; 2, mild invasion into the upper middle zone; 3, moderate invasion well into the middle zone; 4, marked invasion into the deep zone but not to the tidemark; and 5, severe full-thickness degradation to the tidemark. The extent of tibial plateau involvement and proteoglycan loss were scored as 1 (minimal), 2 (mild), 3 (moderate), and 4 (severe).

### Statistical analysis

Statistical analysis was performed using SAS software version 7 (SAS Institute, Cary, NC), and all results are expressed as a means ± SD. The variables that were measured at different time points were analyzed with two-way repeated measures ANOVA with time and group as independent variables and interaction term between time and group. One-way ANOVA was used to assess the metabolic effects of control, curcumin, THC, 17β-estradiol (a positive control), and normal controls (rats with saline injection) at a single time point at the end of the experiment. Significant differences in the main effects among the groups were identified by Tukey’s test at *P* < 0.05.

## Results

### Curcumin and tetrahydrocurcumin suppress menopausal symptoms

**Fig. 2:**
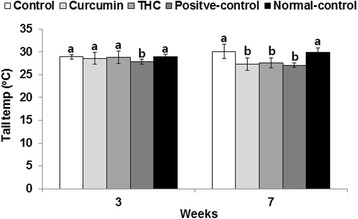
Tail skin temperature at the third and seventh weeks of the experimental periods. Ovariectomized (OVX) rats were provided with a 45 % fat diet containing (1) 0.4 % curcumin, (2) 0.4 % tetrahydrocurcumin (THC), (3) 30 μg/kg body weight 17β-estradiol + 0.4 % dextrin (positive control), (4) 0.4 % dextrin (control), or (5) 0.4 % dextrin (normal control for osteoarthritis). At the beginning of the fifth week, an articular injection of monoiodoacetate into the right knee was performed on all OVX groups except the normal-control group and the assigned diets were provided for an additional 3 weeks. Rats in the normal-control group had articular injections of saline in the right knee. Tail skin temperature was measured at the fourth and seventh weeks. Each bar represents mean ± SD (*n* = 10). (*a*, *b*) The bars with different letters were significantly different among groups by the Tukey test at *P* < 0.05

Tail skin temperature after OVX, representing hot flush in menopausal women, increased over the first 3 weeks in all groups. At the third week, there were few differences in tail temperatures among the groups, with only the positive control having a slightly lower temperature. However, by the seventh week, the curcumin, THC, and positive-control groups all had significantly lower temperatures than the control and normal-control groups (Fig. 2).

OVX rats had higher body weights than positive-control rats at the fourth and seventh weeks, but food intake was not significantly different. Curcumin and THC consumption did not change the increase of body weight in OVX rats, and food intake was not significantly different (Table 1). The average intake of curcumin and THC, as calculated from feed consumption, was about 55–57 mg/day/rat, which corresponds to about 900–1500 mg of each for humans. Peri-uterine and retroperitoneal fat pad weights were greater in control rats than positive-control rats, and unlike body weight, curcumin and THC lowered their fat mass (Table 1). Interestingly, THC decreased the retroperitoneal fat mass more than curcumin. OVX rats had lower uterine weight and serum 17β-estradiol levels, and they were both normalized by 17β-estradiol treatment, but not by curcumin and THC (Table 1). Thus, the modulation of fat mass by curcumin and THC is not due to increased secretion of 17β-estradiol. Serum triglyceride levels also increased in control rats in comparison to the positive-control rats and curcumin, and THC prevented the increase as much as in the positive-control rats.

**Table 1:** Metabolic parameters at the end of experimental periods

	Control (*n* = 10)	Curcumin (*n* = 10)	THC (*n* = 10)	Positive control (*n* = 10)	Normal control (*n* = 10)
Body weight at fourth week (g)	347 ± 32^a^	332 ± 30^a^	337 ± 29^a^	301 ± 22^b^	346 ± 31^a^
Body weight at seventh week (g)	332 ± 34^b^	328 ± 33^b^	329 ± 35^b^	297 ± 26^c^	367 ± 36^a^
Food intake at fourth week (g)	14.8 ± 1.7	14.0 ± 1.5	13.6 ± 2.0	13.1 ± 1.4	14.7 ± 1.8
Food intake at seventh week (g)	13.3 ± 1.6^b^	14.3 ± 1.9^ab^	14.0 ± 1.8^ab^	13.1 ± 1.7^b^	15.4 ± 1.7^a^
Average intake of supplements (mg/day)	–	57.4 ± 6.5	55.6 ± 6.1	–	–
Peri-uterine fat (g)	7.9 ± 1.1^a^	6.1 ± 0.7^b^	6.7 ± 0.8^b^	4.9 ± 0.9^c^	8.4 ± 0.9^a^
Retroperitoneum fat (g)	11.7 ± 2.0^a^	9.9 ± 1.1^b^	7.4 ± 1.0^c^	5.8 ± 0.5^d^	12.3 ± 1.8^a^
Uterine weight (g)	0.17 ± 0.06^b^	0.22 ± 0.07^b^	0.20 ± 0.05^b^	0.51 ± 0.08^a^	0.18 ± 0.05^b^
Serum 17β-estradiol levels (pg/ml)	1.6 ± 0.6^b^	1.8 ± 0.5^b^	1.9 ± 0.5^b^	7.5 ± 1.1^a^	1.7 ± 0.6^b^
Serum triglyceride (mg/dL)	85.9 ± 5.3^a^	71.9 ± 6.4^b^	70.4 ± 7.4^b^	68.3 ± 6.6^b^	84.3 ± 5.5^a^
Serum glucose (mg/dL)	103 ± 8^a^	98 ± 7^a^	100 ± 8^a^	88 ± 10^b^	102 ± 7^a^
Serum insulin (ng/mL)	1.49 ± 0.17^a^	1.21 ± 0.14^b^	1.26 ± 0.17^b^	1.19 ± 0.11^b^	1.43 ± 0.15^a^
HOMA-IR	8.5 ± 0.9^a^	6.6 ± 0.8^b^	6.9 ± 0.9^b^	5.8 ± 0.6^c^	8.1 ± 0.8^a^

Ovariectomized (OVX) rats were provided with a 45 % fat diet containing (1) 0.4 % curcumin, (2) 0.4 % tetrahydrocurcumin (THC), (3) 30 μg/kg body weight 17β-estradiol + 0.4 % dextrose (positive control), (4) 0.4 % dextrose (control), or (5) 0.4 % dextrose (normal control for osteoarthritis). At the beginning of the fifth week, an articular injection of monoiodoacetate into the right knee was performed on all OVX groups except the normal-control group and the assigned diets were provided for additional 3 weeks. Rats in the normal-control group had an articular injection of saline in the right knee. Values are mean ± SD

^a, b, c^Values on the same row with different superscripts were significantly different among groups by the Tukey test at *P* < 0.05

### Glucose metabolism

Control and normal-control rats both had mildly impaired glucose intolerance due to the high-fat diets, as evidenced by HOMA-IR which is a reliable predictor of insulin resistance in rats (Cacho et al. 2008). Overnight-fasted serum glucose levels were higher in both OVX and normal-control groups than in positive-control rats which also had lower serum insulin concentrations (Table 1), indicating increased insulin sensitivity in positive-control rats, which was confirmed by HOMA-IR. Curcumin and THC also increased insulin concentrations and sensitivity, but not quite as much as in positive controls.

**Fig. 3:**
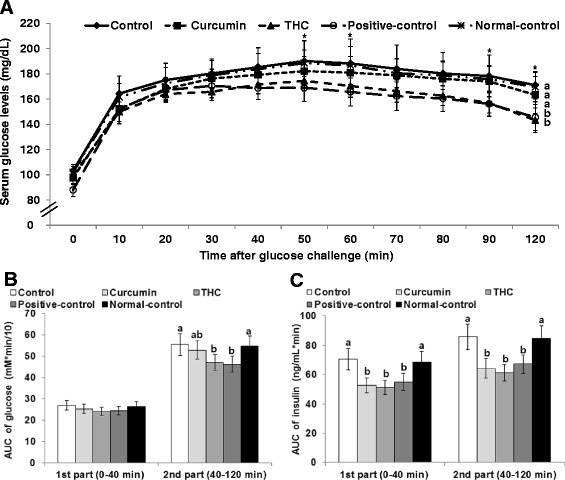
The area under the curve of serum glucose and insulin during oral glucose tolerance test (**a–c**). Ovariectomized (OVX) rats were provided with a 45 % fat diet containing (1) 0.4 % curcumin, (2) 0.4 % tetrahydrocurcumin (THC), (3) 30 μg/kg body weight 17β-estradiol + 0.4 % dextrin (positive control), (4) 0.4 % dextrin (control), or (5) 0.4 % dextrin (normal control for osteoarthritis). At the beginning of the fifth week, an articular injection of monoiodoacetate into the right knee was performed in all OVX groups except the normal-control group. Rats in the normal-control group had articular injections of saline in the right knee. At 2 weeks after injecting MIA, oral glucose tolerance tests were performed with 2 g glucose per kilogram body weight. Blood samples were taken at the time points indicated, glucose and serum were measured, and the area under the curve of glucose and insulin was calculated. The *dots* or *bars* and *error bars* represent mean ± SD (*n* = 10). Asterisks represent the significant treatment effect by repeated measures of a two-way ANOVA test at *P* < 0.05. (*a*, *b*) The bars or dots with different letters were significantly different among groups by the Tukey test at *P* < 0.05

After the oral glucose challenge, serum glucose levels were elevated up to 40–50 min and then they slowly decreased in all rats (Fig. 3a). Control rats had higher peaks at 50 min than the positive-control rats whereas curcumin and THC treatment tended to lower it below that of the normal-control group. Area under the curve (AUC) of glucose in the first and second part of the OGTT was higher in controls than the positive controls, and AUC of glucose was similar in the THC group to the positive control (Fig. 3b). AUC of insulin in the first and second part of the OGTT was much higher in control rats than positive-control rats whereas the curcumin and THC groups had AUC of insulin similar to the positive control (Fig. 3c). Therefore positive-control, curcumin-, and THC-treated rats had greater insulin sensitivities than either the normal-control or control rats.

### Global observations and pain-related behaviors of OA symptoms

**Fig. 4:**
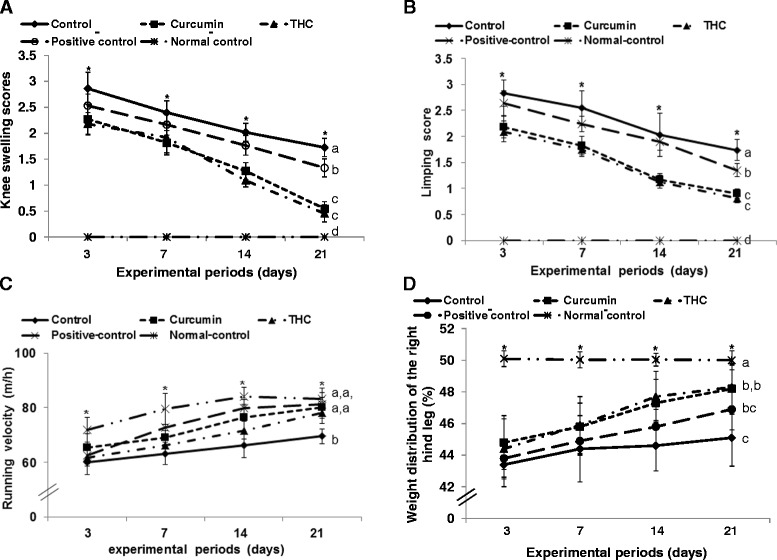
Gross observation of osteoarthritis symptoms and pain-related behaviors at 3, 7, 14, and 21 days after monoiodoacetate (MIA) injection. Ovariectomized (OVX) rats were provided with a 45 % fat diet containing (1) 0.4 % curcumin, (2) 0.4 % tetrahydrocurcumin (THC), (3) 30 μg/kg body weight 17β-estradiol + 0.4 % dextrin (positive control), (4) 0.4 % dextrin (control), or (5) 0.4 % dextrin (normal control for osteoarthritis). At the beginning of the fifth week, an articular injection of monoiodoacetate into the right knee was performed on all OVX groups except the normal-control group and the assigned diets were provided for an additional 3 weeks. Rats in the normal-control group had an articular injection of saline in the right knee. In a gross observation of osteoarthritis symptoms, the scores of the swelling (**a**) and limping (**b**) in the right knee were measured. As indicators of knee pain, differences in weight distribution of the right hind paw (**c**) were measured by an incapacitance tester and maximum running velocity on a treadmill (**d**) was determined. Each *data point* and *error bar* represents the mean ± SD (*n* = 10). *Asterisks* represent the significant treatment effect by repeated measures of a two-way ANOVA test at *P* < 0.05. (*a*, *b*, *c*, *d*) The dots with different letters were significantly different among groups in the Tukey test at *P* < 0.05

MIA, but not saline, injection induced global OA symptoms such as limping and swelling. After MIA injection, the global symptoms were scored weekly. Swelling was greater in the control group than the other groups, and it gradually decreased as time passed. Curcumin and THC markedly reduced swelling to levels near the normal controls by the seventh week, but it was not quite normal (Fig. 4a). In positive controls, swelling was less than in the control but not as low as with curcumin and THC. The limping score showed a similar trend of swelling (Fig. 4b).

OA is accompanied with pain, the severity of which was mainly determined by the maximum velocity of running on a treadmill and asymmetric weight distribution. Running at maximum velocity was slower in the control group, and it increased as time passed, but the increase was small. Rats in the curcumin, THC and positive-control groups had similar maximum running velocities as those in the normal-control group (Fig. 4c). In addition, weight distribution in the right knee was much less than in the left knee since the right knee had pain in all MIA-treated rats during the first week after MIA injection. It was ameliorated better in the curcumin- and THC-treated rats than in the control rats as time passed (Fig. 4d).

### Body composition in the hip and right leg

**Fig. 5:**
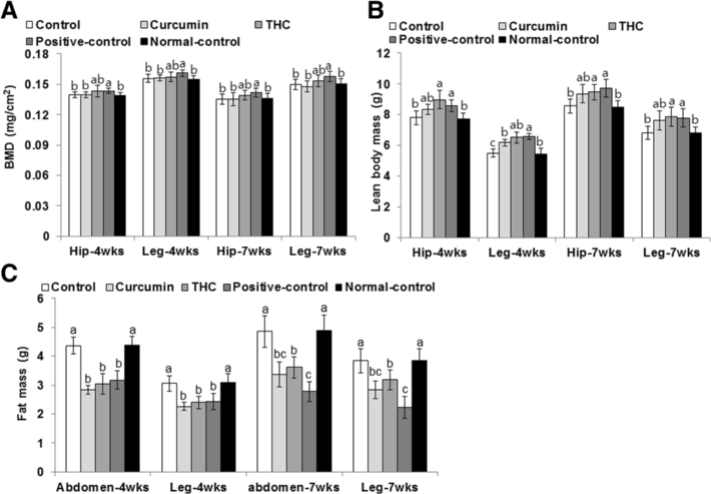
Bone mineral density (BMD) and lean mass of the femur and knee with intra-articular injection of monoiodoacetate (MIA) at days 0 and 21 after MIA injection. Ovariectomized (OVX) rats were provided with a 45 % fat diet containing (1) 0.4 % curcumin, (2) 0.4 % tetrahydrocurcumin (THC), (3) 30 μg/kg body weight 17β-estradiol + 0.4 % dextrin (positive control), (4) 0.4 % dextrin (control), or (5) 0.4 % dextrin (normal control for osteoarthritis). At the beginning of the fifth week, an articular injection of monoiodoacetate into the right knee was performed on all OVX groups except the normal-control group and the assigned diets were provided for an additional 3 weeks. Rats in the normal-control group had an articular injection of saline in the right knee. BMD (**a**) and lean body mass (**b**) of the hip and right leg and fat mass of the abdomen and right leg (**c**) were measured by DEXA. Each *bar* and *error bar* represents the mean ± SD (*n* = 10). (*a*, *b*, *c*) The bars with different letters were significantly different among groups by Tukey test at *P* < 0.05

After 4 weeks of OVX, BMD of the hip and right leg began to be lower in control rats than positive-control rats and continued to decrease through week 7 (Fig. 5a). Curcumin and THC did not prevent the loss of BMD in comparison to the control group. Lean body mass was lower in control rats than positive-control rats in weeks 4 and 7, but unlike BMD, the loss was reversed and it was partially restored by week 7. Curcumin exhibited a tendency to increase lean body mass in the hips and legs, but it was significantly different from other groups only in the right leg at 4 weeks (Fig. 5b). THC increased the lean body mass in the hip and leg to as much as the positive control. The increase in lean body mass was also associated with decreased fat mass. Control rats had much greater fat mass in the hip and right leg than positive-control rats (Fig. 5c). Fat mass was much greater in the control rats than in the positive-control rats in the fourth and seventh week, and curcumin and THC prevented the increase in comparison to the control rats. At the seventh week, the decrease in the hip and leg was not as much as in the positive-control rats.

### The mRNA expressions of cytokines in the articular cartilage of the right knee

**Fig. 6:**
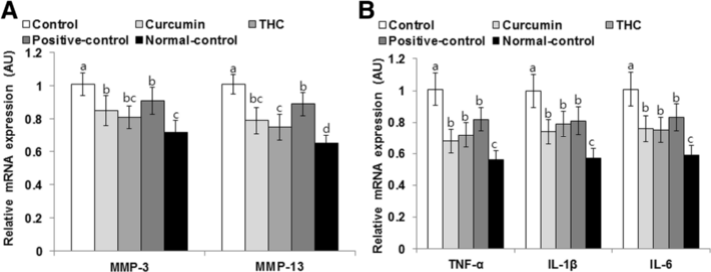
The mRNA expression of matrix metalloproteinases and pro-inflammatory cytokines in the articular cartilage at 21 days after intra-articular injection of monoiodoacetate (MIA). Ovariectomized (OVX) rats were provided with a 45 % fat diet containing (1) 0.4 % curcumin, (2) 0.4 % tetrahydrocurcumin (THC), (3) 30 μg/kg body weight 17β-estradiol + 0.4 % dextrin (positive control), (4) 0.4 % dextrin (control), or (5) 0.4 % dextrin (normal control for osteoarthritis). At the beginning of the fifth week, an articular injection of monoiodoacetate into the right knee was performed on all OVX groups except the normal-control group and the assigned diets were provided for an additional 3 weeks. Rats in the normal-control group had an articular injection of saline in the right knee. The mRNA expressions of MMP3 and MMP13 involved in collage degradation (**a**) and cytokines (TNF-α, IL1β, and IL6) that result in inflammation (**b**) were measured by real-time PCR. Each *bar* and *error bar* represents the mean ± SD (*n* = 6). (*a*, *b*, *c*) The bars with different letters were significantly different among groups by Tukey’s test at *P* < 0.05

MIA is known to initiate OA by activating matrix metalloproteinases and by inducing inflammation and elevating cytokine release. MMPs are collagenases that break down the extracellular matrix. The expressions of *MMP3* and *MMP13* in the articular cartilage increased in more control rats than in normal-control rats, but curcumin and THC suppressed their expressions compared to the control group. THC decreased their expression slightly better than curcumin, but it was not significantly different whereas the positive-control group did not decrease as much as the THC (Fig. 6a).

MIA increased the expression of pro-inflammatory cytokines such as *TNF-α*, *IL1β*, and *IL6* in the articular cartilage of control rats in comparison to the normal-control rats. Curcumin and THC prevented the increase in the pro-inflammatory cytokine expressions in comparison to the control group, and estradiol treatment also decreased their expression (Fig. 6b). Curcumin lowered TNF-α expression more than the 17β-estradiol treatment of the positive controls.

### Histopathological analysis

**Fig. 7:**
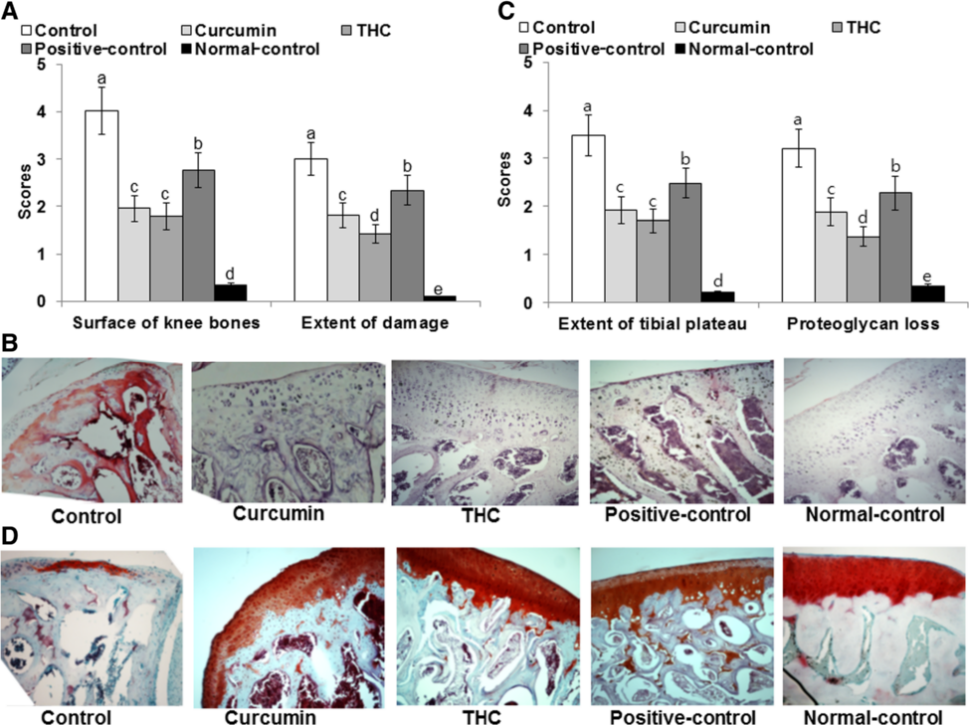
The histopathological features of osteoarthritic lesions in the knee joints of rats at 21 days after intra-articular injection of monoiodoacetate (MIA). Ovariectomized (OVX) rats were provided with a 45 % fat diet containing (1) 0.4 % curcumin, (2) 0.4 % tetrahydrocurcumin (THC), (3) 30 μg/kg body weight 17β-estradiol + 0.4 % dextrin (positive control), (4) 0.4 % dextrin (control), or (5) 0.4 % dextrin (normal control for osteoarthritis). At the beginning of the fifth week, an articular injection of monoiodoacetate into the right knee was performed on all OVX groups except the normal-control group and the assigned diets were provided for an additional 3 weeks. Rats in the normal-control group had an articular injection of saline in the right knee. The depth and extent of cartilage damage and the quantification of the damage (**a**) and histology (**b**) were determined in hematoxylin-eosin-stained, paraffin-embedded knee joint sections from MIA-injected rats (magnifying power ×10). In addition, the depth and extent of cartilage damage and quantification of the cartilage damage (**c**) and histology (**d**) in the MIA-injected knees were evaluated in safranin-O fast green-stained, paraffin-embedded knee joint sections from the osteoarthritic rats (magnifying power ×10). Each *bar* and *error bar* represents the mean ± SD (*n* = 5). (*a*, *b*, *c*, *d*, *e*) Values of the bars with different superscripts were significantly different among groups by the Tukey test at *P* < 0.05

Histological evaluations using H-E staining revealed that MIA injection resulted in damage to the articular cartilage and subchondrial bone of the knee in control rats (Fig. 7a). The normal-control rats exhibited normal articular cartilage structures having smooth articular surfaces, normal chondrocytes with columnar orientation, and intact tidemark and subchondrial bone. However, MIA injection into the knee of OVX rats induced the degeneration of columnar orientation, degeneration of tidemark, and penetration of subchondrial bones, and estrogen deficiency exacerbated the histological scores of the knees injected with MIA (Fig. 7a). In the descending order of 17β-estradiol treatment, curcumin, and THC, the articular structure degeneration was lessened in MIA-injected knees, which reduced the penetration of subchondrial bones, but did not protect against the degeneration of tidemarks (Fig. 7a). Safranin-O fast green staining revealed that proteoglycan loss was increased in control rats whereas the articular cartilage loss was significantly reduced in the descending order of positive-control, curcumin, and THC treatments (Fig. 7b). Curcumin and THC did not improve the morphology of the articular cartilage to similar morphologies as the normal control (Fig. 7b). These results indicate that curcumin and THC treatment prevented the breakdown of the articular surface in OVX rats with the MIA injection into the right knee. THC had a better efficacy in the changes of knee morphology than curcumin in OVX rats injected with MIA.

## Discussion

The present study compared the efficacies of curcumin and THC for preventing postmenopausal and OA symptoms in OVX rats with MIA injection into the right knee. Curcumin and its food source, turmeric, have long histories as both food and medicines. THC is one of the metabolites of curcumin. Curcumin and THC had similar efficacies for skin tail temperature in OVX rats, whereas THC, but not curcumin, prevented impairment of glucose tolerance. Both protected against OA symptoms and pain-related behaviors better than 17β-estradiol treatment in estrogen-deficient rats. They also maintained lean body mass and lowered fat mass as much as 17β-estradiol treatment. The improvements in OA symptoms were associated with decreased expressions of MMPs and pro-inflammatory cytokines. Therefore, curcumin and THC have similar efficacies for OA symptoms and are potential interventions for human osteoarthritis.

As life expectancy increases, the prevalence of OA is elevated, and women have about a twofold higher incidence than men and it is markedly increased in postmenopausal women. There are also many modifiable risk factors for OA including the following: obesity, high-fat diets, physical activity, muscle strength, joint alignment, and bone metabolism (Johnson and Hunter 2014). Thus, the animal model selected for the present study was an estrogen-deficient OVX rat with MIA injected into the knee and fed a high-fat diet. In the present study, we investigated whether curcumin and THC prevented the exacerbation of OA symptoms in OVX rats injected with MIA into the knee and fed a high-fat diet. This animal model was proper for investigating the changes of OA symptoms by herbal treatment.

Although the cause of OA is not known, OA is related to the loss of articular cartilage due to inflammation and protein degradation enzymes. OA treatment focuses on relieving pain, improving joint mobility, increasing the strength of the joints, and minimizing the disabling effects of the disease (Aggarwal et al. 2015). The drugs commercially used have adverse effects, and alternative treatments have been investigated. Curcumin is a highly pleiotropic molecule with a strong safety record. Convincing molecular evidence supports its efficacy for targeting multiple inflammatory diseases including OA. Curcumin has been reported to reduce inflammation in OA in cell-based studies and animal and human studies (Colitti et al. 2012; Henrotin et al. 2010; Panahi et al. 2015; Panahi et al. 2014; Shakibaei et al. 2011; Yeh et al. 2015). Most of these activities have been assigned to methoxy, hydroxyl, α,β-unsaturated carbonyl, or diketone groups present in curcumin (Liang et al. 2009). Several modified curcumins have been synthesized and studied for better efficacy. One of the major metabolites of curcumin is THC, which lacks the α,β-unsaturated carbonyl moiety and is white in color. The change of chemical structure may modify its functionality and efficacy.

Although the cause of OA is unknown, it is highly associated with inflammation. Various enzymes and cytokines such as COX-2, 5-lipooxygenase, TNF-α, IL-1, IL-6, IL-8, IL-17, IL-21, IL-23, and monocyte chemotactic protein-1 (MCP-1) are associated with inflammation (Jain et al. 2009). Among these, TNF-α is a major mediator of inflammation which activates caspase-mediated apoptosis, nuclear factor kappa-light-chain-enhancer of activated B cells (NF-κB), activator protein-1, c-Jun N-terminal kinase, p38, and mitogen-activated protein kinase signaling and leads to cell apoptosis. The production of TNF-α from macrophages is activated by various stimuli such as LPS and MIA (Leys et al. 2013; Orita et al. 2011). Curcumin suppresses the gene expression of *TNF-α*, *IL6*, and *IL8* induced by UVB and LPS, thereby suppressing inflammation (Liang et al. 2009). Pae et al. (2008) has reported that the active site for controlling inflammation is located at the α,β-unsaturated carbonyl moiety of curcumin and that THC is therefore less effective for anti-inflammatory activity. However, it has been reported that curcumin and THC have similar inhibitory effects on TNF-α production, and NF-κB activation in LPS-administered mice (Nishida et al. 2010). However, the production of reactive oxygen species are suppressed better by THC than by curcumin (Nishida et al. 2010). Some studies have even shown that THC provides superior protection against inflammation and apoptosis in animal studies (Song et al. 2015; Wu et al. 2014). Therefore, THC is reported to have some different activities and efficacies with different molecular targets in comparison to curcumin (Aggarwal et al. 2015). However, THC has not been previously studied for relieving OA. The present study found that curcumin and THC had a similar efficacy for preventing the exacerbation of OA by decreasing the expression of cytokines such as *TNF-α*, *IL1β*, and *IL6* in the articular cartilage.

MMP family proteins are involved in the breakdown of the extracellular matrix in normal physiological processes, such as embryonic development, reproduction, and tissue remodeling. These proteins include interstitial collagenases 1 and 3 (MMP1 and MMP13, respectively), gelatinases A and B (MMP2 and MMP9, respectively), and stromelysin-1 (MMP3) (Clutterbuck et al. 2009). MMPs exert these activities regulating the release of growth factors and cytokines. These MMPs are involved in the degradation of proteoglycans and non-helical regions of collagens to develop OA. Excess MMP activity is associated with extracellular matrix destruction in various inflammatory conditions including OA (Clutterbuck et al. 2009). Previous studies have demonstrated that the mRNA and protein of MMP2, 3, 9, and 13 are detected in the tibial plateau cartilage, but only mRNA levels of *MMP1* are detected (Gepstein et al. 2002). Thus, *MMP3* and *MMP13* are involved in collagen degradation, leading to the development of OA (Henrotin et al. 2010; Moon et al. 2013; van den Berg 2011). The present study showed that curcumin and THC decreased the expression of *MMP3* and *MMP13* in the articular cartilage, which may have prevented the breakdown of collagen in the knee. Their mRNA expression and protein levels are tightly regulated by transcriptional, translational, and posttranslational stages (Gepstein et al. 2002; van den Berg 2011). It is better to measure protein levels and activities, but due to the limitation of the samples, only mRNA levels were determined. MMP expression is associated with the catabolic actions of pro-inflammatory cytokines such as TNF-α, IL1β, and IL6 in the articular cartilage of animals and humans, and their expression is increased by stimulating NF-κB signaling and impairing TGF-β signaling. Previous studies have demonstrated that curcumin decreased MMP13 in chondrocytes derived from rabbit and suppressed the NF-ĸB activation via the inhibition of IĸBα phosphorylation (Henrotin et al. 2010; Yang et al. 2013). Thus, not only curcumin but also THC suppressed the degradation of articular cartilage and MMP3 and MMP13 expressions.

OA and diabetes commonly co-exist, and clinical studies have suggested that diabetes may augment the development and severity of OA (King and Rosenthal 2015). The progression of OA is also associated with glucose homeostasis. It is well known that serum glucose levels are positively associated with advanced glycation end products (AGEs) and diabetes increases AGE in the circulation and tissues (Cohen 2013). In addition, AGE in the articular cartilage increases with aging (DeGroot et al. 2001; Yan and Li 2013). Increased AGE in the articular cartilage is one of the possible mediators of OA. Intracellular AGE accumulation in chondrocytes is associated with the occurrence and progression of OA through ER stress (Yamabe et al. 2013). Thus, although we did not determine AGEs in this study, hyperglycemia- and glucose-derived AGEs could contribute to the development of OA (Yan and Li 2013) and should be studied in the future. In the present study, interestingly, THC, but not curcumin, enhanced glucose tolerance after glucose challenge in the present study. This indicated that serum glucose levels may be lower after meals with THC treatment and it may decrease AGE in the articular cartilage. This may delay the initiation and progression of OA in the long term. Therefore, THC might have better effects for long-term treatment of OA than curcumin. Therefore, the duration of the study is one limitation of the study since long-term effects may not have been revealed in this study. Other limitations include the use of animal models since OVX does not fully mimic menopause and MIA injections do not fully mimic the etiology of osteoarthritis. However, both are useful and well-established models. Finally, rodents may not metabolize bioactive compounds such as curcumin and THC in the same ways as do humans. Therefore, the results of this study should be confirmed in human clinical trials.

## Conclusions

Curcumin and THC lessened OA symptoms and pain-related behaviors in estrogen-deficient rats fed a high-fat diet. THC exhibited better changes in MIA-injected knee morphology than curcumin possibly by reducing *MMP3* and *MMP13* expressions. In addition, THC, but not curcumin, improved glucose tolerance, which might indirectly delay the long-term progression of osteoarthritis. THC and curcumin are potential new candidates for treating postmenopausal symptoms and osteoarthritis in humans, and THC appears to have some advantages over curcumin for long-term treatment.
